# UPSIDES Mental Health Peer Support in Face of the COVID-19 Pandemic: Actions and Insights

**DOI:** 10.1007/s10597-022-01030-9

**Published:** 2022-12-12

**Authors:** Yael Goldfarb, Alina Grayzman, Lion Gai Meir, Shimri Hadas Grundman, Meirav Rabinian, Max Lachman, Paula Garber Epstein, Inbar Adler Ben-Dor, Adi Naaman, Bernd Puschner, Galia S. Moran

**Affiliations:** 1 Department of Social Work, Ben Gurion University of the Negev, Be’er Sheva, Israel; 2 Enosh the Israeli Mental Health Association, Kfar Saba, Israel; 3 Yozma Derech Halev Consumers Providers, Kfar Saba, Israel; 4 Department of Community Mental Health, University of Haifa, Haifa, Israel; 5 The Bob Shapell School of Social Work, Tel Aviv University, Tel Aviv, Israel; 6 Mental Health Department, Ministry of Health, Jerusalem, Israel; 7 Department of Psychiatry II, Ulm University, Ulm, Germany

**Keywords:** Mental health peer support, Serious mental illness, COVID-19, UPSIDES, Social inclusion

## Abstract

The outburst of the COVID-19 pandemic challenged vulnerable populations such as individuals with significant mental illness. In this fresh focus, we describe the innovative development of the UPSIDES mental health peer support intervention, in face of the COVID-19 pandemic in Israel. While the research program is still ongoing, in this paper we focus on the processes and lessons learned from dealing with the rapidly changing circumstances of the pandemic. We portray additional activities conducted above and beyond the UPSIDES protocol in order to maintain continuation and prevent dropout. We learned that an essential combination of keeping a close adherence with the core peer principles and UPSIDES’ systematic program and the use of flexible telecommunication means, helped to maintain social connection and service users’ participation throughout these times. The sudden pandemic challenges appeared to level out power imbalances and accelerated the formation of reciprocal and supportive relational interactions within the intervention. These processes highlight experiential knowledge as a unique asset, and peer support services as useful in supporting individuals with significant mental illness throughout COVID-19.

## Background

The coronavirus pandemic (COVID-19) had immense effects worldwide. From direct consequences such as fear of being infected and mourning the loss of loved ones, to secondary effects resulting from the measures taken to contain the pandemic, such as physical distancing regulations, national lockdowns and economic instability. Individuals with Significant Mental Illness (SMI) face disadvantaged circumstances pre-pandemic, and stand out as a group particularly vulnerable to its’ effects (Nieweglowski & Sheehan, 2021). Services which are formulated to address the needs of people with SMI and maintain the continuity of access to mental health supports through these unstable times are therefore imperative.

Peer support, meaning social and emotional support which is offered by an individual in equal standing, was suggested as an asset that can be put to use during the COVID-19 pandemic for managing the impact of the pandemic for healthcare workers on the frontline of the COVID-19 outbreak (Cheng et al., 2020). Peer support was also highlighted as a valuable tool for individuals in the general population who encountered mental health deterioration due to the pandemic (Suresh et al., 2021) .The use of peer support within mental health services for people with SMI has been developed already before the pandemic, and was called upon to help support their needs during COVID-19 (Nieweglowski & Sheehan, 2021). Mental health peer support is based on the premise of using one’s lived experience of psychiatric disorders to provide hope and support to others with similar conditions (Davidson et al., 2012; Salzer, 2002, 2010). Mental health Peer Support Workers (PSWs) support service users by role-modeling that recovery is possible, sharing knowledge from experience and using reciprocal empathic relationships (Puschner et al., 2019; Repper & Carter, 2011).

As the PSW role burgeoned in various Mental Health (MH) systems, a need for scaling-up has been called upon. UPSIDES - Using Peer Support In Developing Empowering mental health Services is an international 5-year project in which an innovative peer intervention was developed, integrating implementation research evaluation within a participatory multi-stakeholder approach. While peer support has been implemented in mental health services in the past, UPSIDES is the first international initiative with rigorous development and a comprehensive research program with potential for harmonizing a generic intervention across diverse socio-economic status, cultures and organizational contexts (Moran et al., 2020; Puschner et al., 2019).

UPSIDES is developed within a six-country consortium in a range of high- (Germany, Israel), lower middle- (India) and low-income (Tanzania, Uganda) settings. The research and implementation team involves scientists, mental health professionals, peer workers, and service users working collaboratively in local and international teams. UPSIDES consortium members connect regularly with multiple modalities, using online meetings, WhatsApp groups, a collaborative website (see www.upsides.org) meetings in international and local conferences and workshops. The primary service user outcome is social inclusion, in line with the core values underlying peer support and recovery. In addition to this main outcome, multiple service user indicators are assessed in UPSIDES as part of an RCT research design. Outcomes address recovery, clinical status, psychosocial status, relational aspects with the peer providers, cost-effectiveness and more. The research project includes evaluation of multi stakeholders (PSWs, mental health staff, directors of the programs and key policy makers involved), employing mixed research methods. Data collection is still ongoing and will be published separately in the future (for a more detailed account of the studies involved see Moran et al., 2020).

The COVID-19 pandemic outbreak occurred just shortly after the onset of the UPSIDES intervention, demanding swift adjustments across all 6 study-sites. In this article we portray our experiences of facing COVID-19 pandemic challenges related to the implementation of UPSIDES in Israel. We hereby provide the background regarding UPSIDES’ project development since 2018 until the outbreak of the pandemic; a description of the state of UPSIDES in the Israeli site prior to the outbreak of the pandemic; a description of the pandemic’s impact on UPSIDES intervention; the actions taken in order to maintain connections throughout lockdowns; resuming to in-person meetings and navigating the intervention along with the ongoing pandemic. Finally, we share the key lessons learned from these events and experiences. These insights can inform other services navigating the needs of people with SMI conditions in similar contexts.

## Development of UPSIDES International Training and Intervention

UPSIDES project’s first phase (2018–2019) involved exerted efforts for a systematic study and rigorous development of the state of the art knowledge in the field in order to develop a MH peer support intervention which will be a culturally appropriate and adaptive to multiple contexts. The developed intervention was thus informed by multiple inputs including: (1) systematic reviews of PSW trainings and organizational influences on the implementation of peer interventions (Ibrahim et al., 2020); (2) previous experience of each consortium partner in delivering peer support trainings and interventions; (3) focus groups and expert panels informing current state and needs at each study site (Puschner et al., 2019); a pilot intervention study (Nixdorf et al., 2022). All of these combined together informed UPSIDES initial intervention and then its modifications (Charles et al., 2021; Nixdorf et al., 2022).

The resulting UPSIDES intervention reflects at its base 8 core peer principles: (1) shared-mutual experiences of PSWs and service users; (2) a reciprocal relationship in which both parts give and receive; (3) a non-directive approach developed together (instead of imposed from one side to the other); (4) a focus on service users in their path to recovery; (5) using a strengths based positive attitude; (6) being inclusive to all service users regardless of the nature of their problems and their beliefs to help them find their place in society; (7) striving to progress together towards recovery and (8) creating a common basis of trust and safety. Overall, UPSIDES training includes 12 standardized modules which can be adapted simultaneously for different contexts. Example core modules are recovery, communication, peer network, activating resources, problem solving and more. The core training can be supplemented by additional modules such as stigma; trauma, or dealing with catastrophe, depending on their relevance to the specific setting and the service users in question, making the training easily adaptable to different study sites.

The finalized UPSIDES intervention version included an implementation package, offering a structured program committed to universal core peer principles, while maintaining cultural and organizational flexibility which allows variances in content and organizational needs existing in each country. UPSIDES can be delivered either one-on-one or in a group format, in medical or community mental health services. For example, in the Israeli site, the UPSIDES intervention was designed into a small group format which was found to be a better fit for the existing organizational structure of community mental health services, while in a site in Germany services are offered one-on-one in a psychiatric hospital. In regard to content, some sites, such as in Israel, emphasize active use of experiential knowledge in the forefront of the intervention while in other sites, PSWs also focus on other aspects of service. For example, in Uganda access to mental health services is limited and PSWs were recruited to answer the pressing need for mental health personnel. They operated as bridge connectors between mental health services and the community living in rural areas. In Tanzania, PSW roles were intended to change attitudes and common folk beliefs about the spiritual origin of mental illness and lend psychosocial support close to home, while keeping in touch with the family. Still, the intervention across all sites is based on a premise of peer principles. Social support and recovery role modelling are central aspects. Peer support is provided without hierarchy or judgement, focusing on providing tangible supports, sharing personal experiences of mental health and ill-health and promoting hope and a sense of control.

In addition, the intervention in not a stand-alone entity. Rather, its’ implementation is essentially supported by *an organizational package* which includes educational activities for the staff of the organization where the intervention is implemented, in addition to peer worker training manual and on-going supervision for PSWs (for more description and publications from the international project see www.upsides.org).

## UPSIDES Training and Intervention in Israel

Two designated services were chosen for the implementation of UPSIDES in Israel: “Kidum Proyektim Shikumiim” & “Enosh”. These services are offered as part of a Rehabilitation in the Community of Persons with Mental Disabilities Law, which was enacted in 2000. Services include a wide range of rehabilitation services and programs supervised by the Ministry of Health including housing, employment, adult education, social and leisure time activity, assistance to families, dental care and case management (Aviram et al., 2012; Ministry of Health, 2022).

The intervention was implemented in small group formats named “Chevruta” (literal translation: ‘friendship’ or ‘companionship’), a term borrowed from the traditional learning practice of Jewish scriptures. Chevruta involves a self-directed dialogue between two or more people focused on understanding and solving scholastic problems which enhances the engagement and autonomy of the participants. A sense of camaraderie and interdependence is at the core of these discussions, creating a space in which debates and controversies can lead to powerful insights (Kent & Cook, 2012; Schwarz, 2018). Camaraderie is core to peer support and expresses the sense of closeness that forms from sharing similar experiences, like brothers in arms do. It is solicited through talking openly about past experiences as something both the PSWs and the service users have in common, but each group member learns to steer his personal journey towards recovery in a subjective manner. Each Chevruta is delivered by 2 UPSIDES trained PSWs over 12 sessions, in a flexible time schedule ranging from 3 to 6 months (coordinated with the participants). *Regardless of the content of each session, the main requirement is that all meetings adhere to values of camaraderie and sharing of lived experience, which are at the basis of the intervention.*

The UPSIDES training was held in the months just prior to the outbreak of the COVID-19 pandemic. Eighteen PSW trainees participated in an 8-day UPSIDES training program carried out in ‘Kidum Proyektim Shikumiim’ community mental health services (funded by the ministry of health, MH rehabilitation department) from November 2019 to January 2020. The training was supported by a consumer-provider organization (Yozma—Derech Halev) another service of the Ministry of Health (Grundman et al., 2020) who partnered with UPSIDES research team and provided organizational and PSW supports. The training course was provided by two senior PSWs (PSWs with lived-experience who also provide supervision for less experiences PSWs), referred herein as PSS—Peer Support Supervisors)—Shimri Hadas Grundman who had taken the UPSIDES international train-the trainer course and Meirav Rabinian—an experienced peer specialist. PSW training candidates underwent initial screening and interviews. Motivation and willingness to self-disclose and openness to use one’s experiential knowledge were a central criterion for being selected. Major health problems or instability negatively impacted the possibility to participate. All trainees had previous work experience in mental health services as consumer-providers—an existent role within MH services in Israel. Both Consumer-providers and peer specialist are individuals with a lived experience of having a mental illness who provide services to others with SMI. However, consumer-providers work roles are combined within traditional mental health roles (e.g., ADL support worker, or rehabilitation instructor) and may sometimes focus on more functional aspects, while sharing their past histories when they feel it is appropriate to their client. As for PSW roles - their job descriptions require the explicit use of experiential knowledge. They mostly support others based on their personal knowledge, and employ their lived experiences (e.g., sharing their recovery story) (Moran, 2018).

The training was carried out according to the key guidelines shared by all UPSIDES international sites described above, meant to promote a feeling of fellowship and camaraderie. The group training program encouraged the participating PSW trainees to establish a peer network enabling them to learn from each other’s knowledge and experience. This resulted in an active WhatsApp group of PSWs (which later turned out to be a central and effective tool in the pandemic outbreaks).

Following the completion of the training on February 2020, eight Chevruta groups were initiated across three different geographical localities. Each Chevruta consisted of three to six service users facilitated by two UPSIDES trained PSWs. The PSWs began participating in regular supervision sessions and each received bi-weekly individual phone-call supervision sessions from the PSSs. However, the pandemic broke shortly after the intervention began with most groups having completed just one or two in-person session. We describe next the line of events, health and social challenges and the measures taken to overcome the COVID-19 pandemic in implementing UPSIDES.

## Implications of COVID-19 on UPSIDES Implementation in Israel

### Initial Reactions to COVID-19 Outbreak: Pausing the Intervention

As elsewhere, Israel was gravelly influenced by the effects of the COVID-19 pandemic, resulting in a national lockdown issued on March 19, 2020 which lasted a duration of 11 weeks, offset only in May. In this first lockdown people were restricted from leaving their home, apart from purposes such as purchasing food or medicine and working in businesses defined as essential. Fines and legal sanctions were a major threat if one did not adhere to the restrictions. Soon-after the international UPSIDES research teams and the local Israeli steering committee convened (via online platforms) for consultation. The group was concerned; UPSIDES’ main outcome—social inclusion, and the heart of the intervention relied on interpersonal connection, reciprocity and camaraderie. All of these were now under risk given these new unprecedented conditions. Participants were distraught by the halting of life in general, the sudden isolation and loneliness that resulted by it, and disappointed about the situation’s negative impact on the ability to continue with UPSIDES intervention.

In the face of the turn of COVID-19’s related events, the recognition that digital health technologies are required in order to maintain the continuity of the UPSIDES intervention were evident. While the use of digital communicating platforms such as telephone conversations, video-conferences, and smartphone applications like WhatsApp has previously been suggested in the field of MH, they were often regarded with ambivalence and some suspicion by service users and staff (Barak & Grohol, 2011; Chamberlain & Smith, 2018; Lopez et al., 2019). Yet, as in other community-based programs, UPSIDES’ team found that adapting digital health to the needs of individuals with SMI was no longer a supplementary mean of service or luxury but rather a pressing need (Torous & Keshavan, 2020), especially if one is to maintain social connections while in physical distancing (Kozloff et al., 2020). Yet it wasn’t clear to the UPSIDES team how exactly and to what degree would using such technologies would be effective. The international UPSIDES consortium came to a decision to halt all intervention activities across sites, not allowing the possibility of an online format for Chevruta meeting. This decision was informed by service users who opted for pausing the intervention until in person meetings were possible again. In Israel, the few Chevruta meetings held before the lockdown were helpful in that they’ve already developed an initial supportive relationship among its participants and an appetite for more. Participants expressed curiosity and expectation about UPSIDES. One participant described these meetings as a ‘promo’, with much eagerness for what comes up in the next meetings. Since Chavrutas were convened in small groups, the team expected they could resume in a relatively short time.

### Maintaining Social Connections Throughout Lockdowns

Although in-person group meetings were paused all together, the UPSIDES teams (researchers, MH staff, PSWs and service users) remained connected. Remote-communication channels were used with service user participants of the Chevrutas, with PSWs and their supervision sessions as well as with organization members and international team. The continuous connections, conversations and interactions were highly supportive allowing to vent the situation, readjust and plan ahead. These connections among all UPSIDES stakeholders created a kind of network and relational chain for supporting PSWs and Chevruta participants compromised mental health states due to the pandemic and the consequences of the strict lockdown. These connections were also essential in maintaining the intervention momentum from dissipating until it resumed. The online/phone connections and their characteristics are described in more detail next:

#### With Chevruta Participants

PSWs held regular weekly one-on-one phone conversations with the service users according to a scripted conversation provided by the international UPSIDES consortium due to the pandemic. The purpose of the phone call was to maintain connection and prevent dropout, without resuming the intervention itself. Therefore, PSWs were guided to refrain from referring to UPSIDES intervention content, but conduct a short telephone conversation, expressing general interest and care. In these calls PSWs expressed interest in the feelings and activities of the Chevruta participants, and inquired about possible ways to support their well-being. PSWs also provided up-to-date COVID-19 related information, helping participants follow-up on the rapidly changing reports and guidelines. These phone calls were well received. They were regarded as offering stability and ongoing support in turbulent times, promoting feelings of hope and encouragement. One Chevruta participant described her feelings during these weeks as filled with anxiety and depression leading her to push most people away. The regular conversations with the PSW, regarded in the unique position of a peer who reaches out continuously ‘from the outside’ (of the regular MH staff) provided a sense of support and stability and gave her an opportunity to share her feelings and receive information. PSWs also referred to these calls as providing a sense of control, saying that “specifically on days of lockdown with limited opportunities to meet and receive social support, the calls I made to service users and making use of experiential knowledge gave us a sense of competence… and a feeling we are not alone”.

#### With PSWs

PSSs facilitated bi-weekly supervision sessions on a video-conference platform to support PSWs in this state and help with their interactions with Chevruta participants over the phone calls. The PSWs were experiencing the dire impact of social isolation which served to enhance their motivation to participate in the supervision group meetings, sharing their feelings. Notably, PSSs encouraged the PSWs to jointly explore their coping strategies in time of crisis which are rooted in their own lived experiences, and co-created a list of useful tools. These understandings permeated to the service users who through the phone conversations held with trained UPSIDES PSWs, came to acknowledge their life experiences as powerful assets. Such multi-layered processes in which the experiences of the supporters and the supported are intertwined in reciprocal empathic relationships are at the heart of the peer principles and values (Davidson et al., 2012; Puschner et al., 2019). Interestingly, the online platform served as a leveling arena, where space for intimacy was enhanced in face of the pandemic. Technical issues were also discussed and processed.

#### Staying Connected and Creative with UPSIDES Project

Continuing international online meetings, and accelerating media production related to the project, intensified online communication among the broader tram of UPSIDES. These involved higher frequencies of online meeting of PSSs with UPSIDES research coordinator, involvement in developing a unique Covid related UPSIDES newsletter which was distributed to all sites and on the website (https://www.upsides.org/category/newsletter/) and enhanced accessibility of the project investigators to interested stakeholders.

### Resuming the UPSIDES Intervention

After an 11 weeks break, in-person Chevruta meetings were resumed in May 2020 in a gradual process. At that time, the official lockdown measures were withdrawn and in-person group meetings were allowed in accordance with the ministry of health guidelines (e.g., yet restricted by number of attendees and with a mask requirement). It was challenging and required unique intervening both with PSWs and the staff at the ‘Kidum Proyektim Shikumiim’ organization. While most of the service users were excited about resuming the in-person meetings, PSWs’ excitement was dampened by fear and self-doubts; questioning how they can deal with absenteeism? how to keep health guidelines? And how to deal with changes again after getting used to a ‘new normal’? Reconnecting felt risky and surreal, and returning to in-person group meetings seemed unreasonable, leading to more fear and insecurity.

This led PSSs to use supervision sessions to reflect on PSWs emotions, share their own currently related personal dilemmas and uncertainties and apply coping strategies employing a reciprocal approach. They intentionally encouraged PSWs to find answers within themselves and explore their own experiential knowledge relevant to the fears and questions elicited, rather than look for explicit answers from the PSS. One of the service users portrayed the personal meaning Chevruta meeting held for him during these times: “The Chevruta mainly helped me feel less despaired… to hold on to hope. As the group meetings continued, I felt that in a time as difficult and challenging as the coronavirus pandemic I am receiving something that I especially need – company, support, and someone who is listening”. In parallel, the UPSIDES team coordinated with staff in the ‘Kidum Proyektim Shikumiim’ service their expectations and secured an appropriate physical environment in which the group meetings were to be resumed. Following this, all 12 Chevruta meetings were carried out in person without further disruptions.

### UPSIDES Online Training Course

At this stage of time, another UPSIDES training course for PSWs was to be hosted by ‘Enosh’ community MH organization (also under the supervision and funding of Ministry of Health). The training was held between September and November 2020. While an online training was already part of the research-project protocol for UPSIDES (Puschner et al., 2019) it now also provided a solution for the COVID-19 epidemic consequences. The possibility of on-line training was also beneficial since the participants were geographically distanced and were able to save time and travel costs.

During the training another national lockdown was issued due to an additional COVID-19 outbreak. Fortunately, it did not affect the training course thanks to the online format. The training was led by two PSSs of which one (Lion Gai Meir) was trained in UPSIDES train-the-trainer international program and has herself accumulated many years of experience in training PSWs in Israel in medical and community MH settings. She was employed in ‘Enosh’ in order to bring knowledge-from-experience into the organization. The on-line-training included practical applications and guidelines which are necessary in order to maintain proper telehealth services (Lopez & Schwenk, 2021; Simpson et al., 2021). Some examples are: the issue of balancing self-disclosure with a sense of personal boundaries, along with pragmatical matters such as keeping the video-camera open, smoking, eating or drinking, privacy considerations, and appropriate seating. Sessions were relatively short with frequent breaks and included a combination of activities and exercises to encourage active participation; exercises were held in small meeting-rooms meant to enhance personal connections, intimacy and self-expression; humor was also integrated in the meetings in order to balance difficult contents and emotional intensity involved when conversing about MH lived experiences. In addition, the WhatsApp group served as a convenient outlet for sharing thoughts and assignments among course trainees.

A great deal of thought was put into *interpreting UPSIDES values into the video-conference setting*. The PSSs took an active role in order to enhance the feeling of equality and avoid hierarchy, emphasizing sayings reflecting core peer orientation such as “this worked for me, but it might not be relevant for you”. Efforts were made to highlight the shared characteristics among group members, but also to recognize the unique coping strategies and personal choices of each individual.

After the training course, this group of trained PSWs carried out an in-person UPSIDES intervention in which eighteen service users participated in Chevruta groups. The 12 Chevruta meetings began on November 2020 till June 2021. Due to a third lockdown which lasted between December 27 to February 7, the intervention was paused for 6 weeks and restarted towards the end of February 2021. During this time, supervision meetings of PSSs and PSWs were held once every two weeks in an online format, and phone conversations were held with participants, following the same modality of the ‘Kidum Proyektim Shikumiim’ service with UPSIDES intervention. Following that, the UPSIDES intervention once again resumed successfully. The following quote by one of the participating PSWs offers a vivid portrayal of the positive effect of the intervention: “I feel that I am a pioneer on a mission. For me it is an opportunity to provide strength and meaning to another person… I am open to tell my story after years of silence, in hope that my words will give the courage to open up, to receive help, to rise from the suffering… when the pandemic broke out, I reminded myself that I know something about life-crisis and that the most important part is support, conversation, not staying alone”.

## Lessons Learned

In the above descriptions about the UPSIDES peer support interface with the COVID-19 pandemic and its aftermath, we found several elements that helped overcome challenges and risk of dropout, and at the same time *gain more momentum for the value of MH peer support* in services. The elements include: adhering to peer principles, UPSIDES’ systematic program, maintaining contact, developing a flexible approach to means of communication. Upon the stable foundations of the UPSIDES core principles and program, innovation and creativity were made possible. These elements allowed to use online technology as a kind of *amplifier of peer principles*, leveling out power imbalances and forming reciprocal and supportive relational interactions.

Importantly, an ongoing and highly interactive collaboration and conversations between PSS, PSWs, MH staff, directors and the research team were essential in overcoming the many challenges faced by the pandemic to the UPSIDES project. These kinds of collaborative relationships involved joint thinking about challenges, dilemmas and questions arising during the UPSIDES intervention and its adaptation in light of the pandemic. In reality this meant investing additional time for joint (often online) meetings. UPSIDES *systemic processes involving training, supervision and implementation* allowed to maintain a space in which three ‘generations’ of individuals with SMI (i.e., PSSs, PSWs and UPSIDES service user participants) could reciprocate and acknowledge their lived experience as a unique asset and source for coping. *Maintaining contact* at all levels including PSS supervision meetings and PSWs keeping in touch with service users, as well as providing support from USPIDES coordinator to PSS - all nurtured feelings of solidarity, mutuality and enhanced the social connections.

Participating in an *online supervision peer-group* appears to be a favorable result of the pandemic limitations. The supervision group can help PSWs to maintain connection with supportive figures who do not necessarily work in the same physical space or even the same service, and increase opportunities for personal and occupational growth, both considered as facilitators for successful integration of PSWs (Han et al., 2021). Indeed online MH peer supervision can help with an already known lack of supervision which curtails the sustainability of PSW roles in MH organizations (Gates et al., 2010; Siantz et al., 2018). It is a convenient possibility, overcoming limitations of time and geography, being more accessible thus more feasible. A further understanding of the implications of virtual vs. in-person communication in the field of mental health peer support is necessary in order to promote effective training and supervision processes.

The accelerated development and use of telehealth services is one of the silver linings of the COVID-19 pandemic with the UPSIDES intervention. By carefully planning and communicating about the selective transitioning to telehealth services while adhering to UPSIDES values helped preserve the advantages of the UPSIDES intervention and maintain social inclusion of its’ recipients. The initial positive accounts of UPSIDES teams and recipients support the feasibility of tele-health supports called for elsewhere (Ben-Zeev, 2020). These changes do not just involve a technical shift, but also *a more flexible approach to service provision*, combining all sorts of means of communication, be it online platforms, social media or simple phone calls.

## Conclusion

The COVID-19 pandemic had a high impact on the general public in terms of curtailment of one’s state of mental health (Maulik et al., 2020). At the same time, feelings of confusion, despair, shame and anxiety are not unfamiliar to individuals who continually have severe mental health issues and experience personal crises. In face of the pandemic outbreak, UPSIDES peer intervention served as a powerful and sustainable support buffering sudden and traumatic challenges and further teaching us about the unique value of the peer support MH model.

A common barrier to establishing peer support in recovery-oriented mental health services is the power imbalance between individuals managing mental health issues and service providers (Mead & Filson, 2017). Challenges in implementation of the peer role in MH services are ongoing (Mahlke et al., 2014; Repper & Carter 2011). They derive (among other things) from the inherently different characteristics embedded in peer roles vs. traditional mental health services. While peer roles emerged from self-help groups based on one’s lived experience and mutuality, traditional MH roles derive from scholastic and clinical models (Davidson et al., 2012; Moran, 2017, 2018; Salzer, 2010). This power imbalance and related challenges can potentially increase during times of social distancing and diminished access to mental health services. However, for the most part, the implementation of UPSIDES during COVID-19 so far was experienced as an event which impacted *all* stakeholders involved in the UPSIDES project, and helped level more the power imbalance. The essence of work via a peer support model gained a center-stage through the enhanced collaborative work in-between PSS, PSWs and staff and research team. A peer value-based approach was embedded in this process when using the digital platforms. The implementation of UPSIDES teaches us of the need to continuously examine what optimal characteristics are needed in the organization in order to meet up to the unique role of peer providers. Together these will allow to enjoy the peers’ contribution across all stakeholders: service users, staff, directors, employers and PSWs themselves.

## Data Availability

Data sharing not applicable as no datasets were generated and/or analyzed for this study
